# An Optical Diffuse Reflectance Model for the Characterization of a Si Wafer with an Evaporated SiO_2_ Layer

**DOI:** 10.3390/s19040892

**Published:** 2019-02-21

**Authors:** Artur Zarzycki, July Galeano, Sylwester Bargiel, Aurore Andrieux, Christophe Gorecki

**Affiliations:** 1 Advanced Materials and Energy MatyEr Research Group, Biomaterials and Electromedicine Laboratory, Instituto Tecnológico Metropolitano, Calle 54A No. 30-01, Medellín 050013, Colombia; julygaleano@itm.edu.co; 2 FEMTO-ST Institute, CNRS UMR6174, University of Bourgogne Franche-Comté, 25030 Besançon, France; sylwester.bargiel@femto-st.fr (S.B.); aurore.andrieux@femto-st.fr (A.A.); christophe.gorecki@femto-st.fr (C.G.)

**Keywords:** optical diffuse reflectance model, model inversion, silicon wafer, silicon dioxide, thickness measurement, thin-film

## Abstract

Thin films are a type of coating that have a very wide spectrum of applications. They may be used as single layers or composed in multilayer stacks, which significantly extend their applications. One of the most commonly used material for thin films is silicon dioxide, SiO_2_. Although there are other tools that can be used to measure the thickness of SiO_2_ films, these tools are very complex and sophisticated. In this article, we propose the use of an exponential two-layer light-material interaction model, throughout its diffuse reflectance spectra, as an alternative for the measurement of the thickness of evaporated SiO_2_ on Si wafers. The proposed model is evaluated experimentally by means of a 980-nm-thick SiO_2_ layer evaporated on a Si wafer. The results show that the proposed model has a strong correlation with the thickness measurements obtained using commercial equipment.

## 1. Introduction

Silicon dioxide, SiO_2_, is one kind of dielectric coating that is widely used in optical multilayer stacks, for covering the losses caused by low absorption and low scattering [1,2,3]. However, the stack could be rendered useless if the optical properties of the SiO_2_ layer are not controlled. One of the most important parameters for ensuring good optical quality in SiO_2_ layers is its density. This can be controlled by using different deposition techniques, e.g., thermal oxidation, plasma-enhanced chemical vapor deposition (PECVD), physical vapor deposition (PVD) and ion beam deposition (IBD), among others [4,5,6,7]. However, regardless of which process is used, another very important—perhaps even the most important—parameter that should be carefully verified is the thickness of the deposited layer.

A number of tools can be used to measure the thickness of deposited SiO_2_. Among those tools, there are those based on mechanical techniques such as a stylus profiler, as well as those based on optical techniques such as ellipsometry or diffuse reflectance spectroscopy [8]. In the case of diffuse reflectance spectroscopy, a beam of white light illuminates a point on the surface of a substrate with a deposited SiO_2_ layer, while the diffuse reflectance spectrum is detected through a spectrometer. This spectrum is later processed through techniques based on Fresnel formalism and scalar scattering theory in order to obtain the thickness of the SiO_2_ material [9].

In this article, we propose the use of an exponential two-layer light-material interaction model as an alternative for the measurement of the thickness of evaporated SiO_2_ on a Si wafer, through its diffuse reflectance spectrum. This type of model is linked with multispectral imaging systems (MSI) and has been widely used, e.g., in biology for the analysis of human skin and the diffuse reflectance of other types of tissues [10,11]. 

The main advantage of the MSI-based system over those previously mentioned systems, such as an ellipsometer or an optical interferometer, which by definition are point measurement systems, is its versatility. For instance, once data are acquired by means of an MSI-based system (e.g., an image of a small part of the wafer or an image of the full wafer), they can be processed in any manner using the model proposed in this article—by processing one single point through the set of points until the map of the whole wafer is processed. Additionally, once data are acquired by means of an MSI-based system, an analysis can be performed at any moment, without having to repeat the data acquisition. From this point of view, point measurement systems are limited—a point, points of interest or mapping must be defined before a measurement can be obtained. Moreover, any new measurement requires both the sample and the equipment. Multispectral imaging systems also seem to have the advantage of having cheaper equipment costs. Another advantage of the proposed method is that it can determine not only one parameter (i.e., thickness), but a few others—such as parameters related to particle size and their concentration, among others that are described in this article.

The proposed model is evaluated in a Si wafer with a 980-nm-thick layer of evaporated SiO_2_, in order to prove that this model can be used in further developing vision-based tools for the analysis of this type of material. This article is divided into the following sections: Section 2 presents the preparation of the Si wafer with the evaporated SiO_2_ layer that was used in the experiment, together with the two-layer light-material interaction model; Section 3 presents the results of this study; Section 4 presents the conclusion. 

## 2. Materials and Methods

Our mathematical thickness model was experimentally verified. For that experiment, we used a silicon wafer with a SiO_2_ layer that was deposited through the evaporation technique and an automated film thickness mapping system, F50-EXR, from Filmetrics [12].

### 2.1. Si Wafer with Evaporated SiO_2_ Layer

As a substrate for the SiO_2_ film deposition, we used a standard double-side polished silicon wafer. The wafer was obtained from monocrystalline silicon, grown by the Czochralski process. The wafer had a diameter of 100 mm, a thickness of 390 nm, a crystallographic orientation of (100) and its type of electrical conductivity was N (phosphorus dopant). 

Before the deposition of the SiO_2_ layer, the wafer was cleaned in an acetone bath, then in ethanol and finally rinsed in DI water. 

Since we wanted to obtain a layer of SiO_2_ that was up to 1 µm thick, we decided to use an evaporation technique in order to deposit it. However, the limitations of the equipment prevented us from obtaining such thick layers in a single-step process, so the process was divided into three steps. Each step was composed of two procedures: (a) the cleaning/activation of the surface, using an ion-beam gun in Ar gas, and (b) the evaporation of SiO_2_ from a target melted by an e-beam gun.

### 2.2. Diffuse Reflectance Model

The light-material interaction model for diffuse reflectance analysis in a semi-infinite turbid medium can be expressed by the exponential formulation presented by Equation (1) [13]:

(1) $$R_{t}=2\mu_{s}^{\prime }\int_{0}^{\infty }e^{-2\left(\mu_{s}^{\prime }+\mu_{a}\right)Z}dZ,$$

where $\mu_{s}^{\prime }$ is the reduced scattering coefficient, $\mu_{a}$ is the absorption coefficient and Z is the thickness of the material.

By extending Equation (1) into two layers, in order to analyze the case of a Si wafer with evaporated SiO_2_, Equation (2) is obtained:

(2) $$R_{t}=2\mu_{s1}^{\prime }\int_{0}^{Z_{1}}e^{-2\left(\mu_{s1}^{\prime }+\mu_{a1}\right)Z}dZ+\;2\mu_{s2}^{\prime }\int_{Z_{1}}^{\infty }e^{-2\left(\mu_{s2}^{\prime }+\mu_{a2}\right)Z}dZ.$$

By solving Equation (2), the following equation, Equation (3), is obtained:

(3) $$R_{t}=-\frac{\mu_{s1}^{\prime }}{\mu_{s1}^{\prime }+\mu_{a1}}\left[e^{-2\left(\mu_{s1}^{\prime }+\mu_{a1}\right)Z_{1}}-1\right]+\frac{\mu_{s2}^{\prime }}{\mu_{s2}^{\prime }+\mu_{a2}}\left[e^{-2\left(\mu_{s2}^{\prime }+\mu_{a2}\right)Z_{1}}\right],$$

here, $R_{t}$ is the total diffuse reflectance obtained from the contribution of absorption and the scattering of light in a Si wafer with evaporated SiO_2_ layer; $Z_{1}$ is the thickness of the first layer, representing the evaporated SiO_2_ layer; $\mu_{a1}$ and $\mu_{s1}^{\prime }$ are the absorption and scattering coefficients of SiO_2_, while $\mu_{a2}$ and $\mu_{s2}^{\prime }$ are the coefficients of the Si wafer.

**Figure 1:**
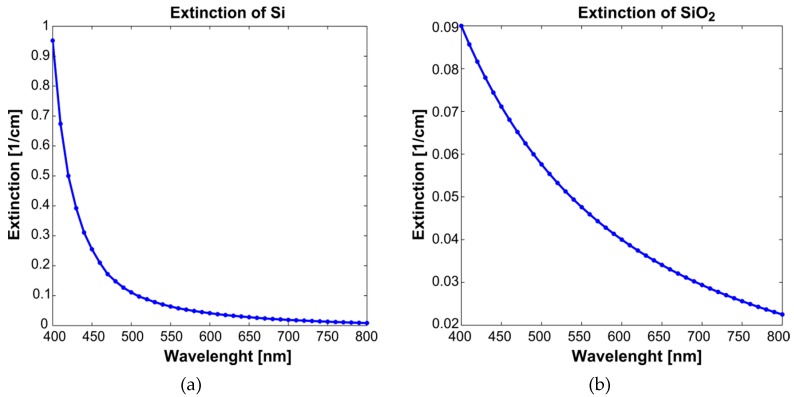
(**a**) The extinction coefficients of Si. Images adapted from Reference [14]. (**b**) The extinction coefficients of SiO_2._ We adapted these images from Dobrowolska et al. [15].

The absorption coefficients of Equation (3) can be expressed as follows:

(4) $$\mu_{a1}=a_{si}\varepsilon_{si},$$

(5) $$\mu_{a2}=a_{SiO_{2}}\varepsilon_{SiO_{2}},$$

where $a_{si}$ and $a_{SiO_{2}}$ are two parameters with normalized values between 0 and 1, which represent the concentrations of Si and SiO_2_ in the evaporated wafer. The components $\varepsilon_{si}$ and $\varepsilon_{SiO_{2}}$ are the extinction coefficients of Si and SiO_2_, as presented in Figure 1a,b, respectively [14,15].

The scattering coefficients were evaluated by means of the Mie theory [16]. This theory calculates the light-scattering of the material, based on a set of variables that might represent the following parameters: the diameter of the particles composing the material (P*_D_*), its volume fraction (P*_VF_*) and the corresponding refractive index (n) of the evaluated material. The parameter related to the diameter of the Si particles, P_D_Si_, is considered to be in the order of 1 nm [17], while the parameter corresponding to the diameter of the particles of SiO_2_, P_D_SiO2_, could be in the order of 100 nm [18]. The refractive index (n) for SiO_2_ is considered to be 1.44, while that for Si is considered to be a wavelength-dependent value, as presented in Reference [12].

### 2.3. Inversion of the Model

The diffuse reflectance spectrum of the Si wafer, as described in Section 2.1, was obtained by means of the F50-EXR system from Filmetrics. This measured spectrum ($R_{m}$) was processed through an inverse-modeling procedure in order to obtain the corresponding parameters described in Section 2.2. These parameters included the concentrations of Si and SiO_2_, $a_{si}$, $a_{SiO_{2}}$ respectively, as well as the thickness of the evaporated SiO_2_ $Z_{1}$.

The implemented inverse-modeling procedure is based on an optimization approach and the two-layer diffuse reflectance model is presented in Section 2.2. The optimization approach used in this work corresponds to the Nelder–Mead simplex algorithm as described by Lagarias et al. [19]. The steps for the inverse-modeling procedure are the following:

1. The variables of the light-material model were initialized in random values.
2. Then, with the current value of the model’s variables, a simulated diffuse reflectance spectrum ($R_{s}$) was calculated by means of Equation (3).
3. After that, the Mean Square Error (MSE) between the simulated reflectance and the measured one was calculated by means of the following equation:
  (6) $$MSE=\frac{1}{n}\sum_{\lambda =400}^{800}{\left(R_{m}-R_{s}\right)}^{2}.$$
4. Once the MSE was evaluated, it was evaluated whether this value converges to a minimum value. If so, the inverse-modeling procedure was ended successfully and the final result corresponds to the current values of the light-material model’s variables. If otherwise, the procedure continued to step 5.
5. In this step, the Nelder–Mead simplex optimization approach calculated new values for the light-material model’s variables. Once these new values were obtained, the procedure returned back to step 2.

## 3. Experimental Results and Discussion

**Figure 2:**
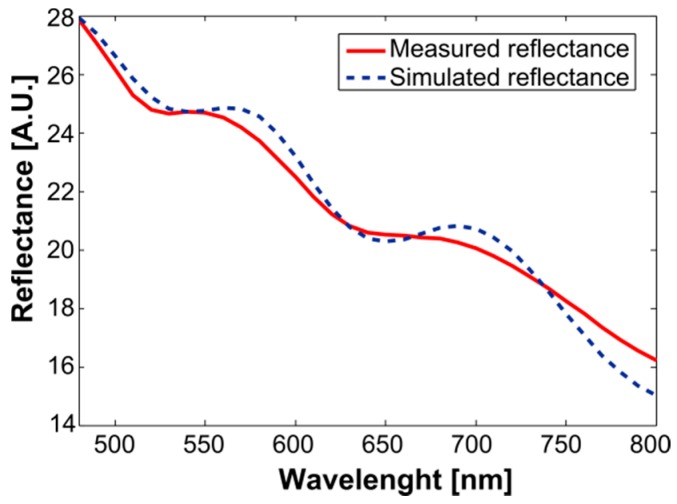
Results obtained from the inverse-modeling procedure. The red line corresponds to the measured diffuse reflectance spectrum ($R_{m}$) of the Si wafer with an evaporated SiO_2_ layer, as described in Section 2.1. The blue line presents the corresponding simulated diffuse reflectance spectrum ($R_{m})$, obtained with the inverse-modeling procedure that is presented in Section 2.3.

In Figure 2, the red line corresponds to the measured diffuse reflectance spectrum ($R_{m}$) of the Si wafer with an evaporated SiO_2_ layer, as described in Section 2.1. The blue line corresponds to the simulated diffuse reflectance spectrum ($R_{m}$), obtained through the inverse-modeling procedure that is presented in Section 2.3. It is possible to observe the congruence between both spectra in Figure 2, indicating that the model obtained a good approximation of the evaluated data. The corresponding results that were obtained for the model’s variables are presented in Table 1. The MSE was 0.32, with a correlation of 0.99 between both spectra.

The values for the thickness of SiO_2_, presented in Table 1, were corroborated with the measurements obtained from the Filmetrics system. The value obtained from the inverse-modeling procedure was the same order of magnitude as the minimum value obtained with the commercial system that was used to measure the thickness of the evaporated SiO_2_ (932.71 nm). Also, the values that were obtained for the parameters that might represent the diameters of the Si and SiO_2_ particles were the same order of magnitude as those that were reported in the literature [17,18].

## 4. Conclusions

In this article, we propose the use of an exponential two-layer light-material interaction model as an alternative tool for measuring the thickness of evaporated SiO_2_ on a Si wafer, through its diffuse reflectance spectra in the visible-near infrared region (VIS-NIR). Diffuse reflectance can be obtained from a spectroscopic-based system. The proposed model is based on the following parameters: the diameters of the Si and SiO_2_ particles, their refraction indices, their extinction coefficients and the thickness of the evaporated SiO_2_ layer. By using an inverse-modeling approach based on the Nelder–Mead simplex optimization algorithm, a diffuse reflectance spectrum of an evaporated Si wafer was processed in order to obtain the values of the corresponding parameters. As a result, the value obtained for the thickness of the SiO_2_ layer was similar in its order of magnitude to the one measured with a commercial system (930 nm when measured with the proposed model vs. 932.71 nm when measured with the commercial system). Also, the modeled spectra had an MSE error of 0.32 and a correlation of 0.99, with respect to the spectra obtained from the commercial system. 

Since exponential two-layer light-material interaction models are widely used for the analysis of the diffuse reflectance spectra of biological tissue, obtained from multispectral images, the results of this article can be used in furthering the development of vision-based tools (such as multispectral systems) for the analysis of Si-based materials. Multispectral systems are used in the analysis of, for example, biological tissue due to the advantage that these systems offer in terms of the field of view: a larger collection of spectral data, concerning areas of interest that exceed the scope of the particular material under evaluation, can be acquired and then analyzed at once by means of this type of exponential model.

## Figures and Tables

**Table 1:** Results of the model’s variables obtained from the inverse-modeling procedure.

Thickness of SiO_2_ (nm)	P_VF_SiO_2__ (%)	P_D_SiO_2__ (nm)	a_SiO_2__ (A.U.)	P_VF_Si_ (A.U.)	P_D_Si_ (nm)	a_Si_ (A.U.)
930	0.20	104	0.34	1.24	1.01	1.14
